# Supplementary material for: Association between a common immunoglobulin heavy chain allele and rheumatic heart disease risk in Oceania
Source: Nat Commun. 2017 May 11;8:14946. doi: 10.1038/ncomms14946 (PMC5437274; doi:10.1038/ncomms14946)
Supplement: Supplementary Information — Supplementary Figures and Supplementary Note [file ncomms14946-s1.pdf]

Supplementary Note

Supplementary Note 1 | Origin of the *IGHV4-6109 allele**

To determine whether the novel allele had been amplified from the *IGHV4-61* or *IGHV4-59* locus, we sequenced three individuals with each *IGHV4-61* haplotype using the amplification primers (Supplementary Fig. 8a). Separate reactions were used for forward and reverse strand primers targeting a 1599 bp product, with each primer amplifying approximately 800 bp of sequence. We then aligned these sequences with the corresponding 473 bp sequence from the main sequencing experiment (Supplementary Figure 11). Across 1,553 sequenced positions, there was noticeably greater identity between the *IGHV4-61**09 sequence and the two previously validated *IGHV4-61* alleles (97.7% identity for *IGHV4-61**01; 97.2% for *IGHV4-61**02) than between *IGHV4-61**09 and *IGHV4-59**01 (94.7% identity), which is the only validated allele at the *IGHV4-59* locus[14]. Moreover, focusing on the 1,013 non-coding positions, there was only one non-coding position at which *IGHV4-61**09 matched *IGHV4-59**01 but not *IGHV4-61**01 or *IGHV4-61**02 (0.1% positions) compared to 67 non-coding positions at which *IGHV4-61**09 matched *IGHV4-61**01 or *IGHV4-61**02 but not *IGHV4-59**01 (6.6% positions).

Supplementary Figures

A

	Fiji n = 598*	New Caledonia n = 492†	Samoa n = 70
Phenotypic sex, n (%)			
Male	215 (36.0)‡	167 (33.9)	26 (37.1)
Female	324 (54.0)‡	325 (66.1)	44 (62.9)
Age, mean (SD)	34.5 (16.2)	39.3 (18.1)	11.5 (2.6)
Severity§, n (%)			
Mild	74 (12.4)	70 (14.2)	25 (35.7)
Moderate	294 (49.2)	197 (40.0)	24 (34.29)
Severe	230 (38.5)	225 (45.7)	21 (30.0)
Mitral stenosis§, n (%)	253 (42.3)	212 (43.1)	10 (14.3)

*21 cases from Fiji and †6 cases from New Caledonia were of insufficient concentration to genotype. ‡59 Fijian cases (9.9%) had missing phenotypic sex due to a database error. §Based on echocardiographic findings. SD, standard deviation.

B

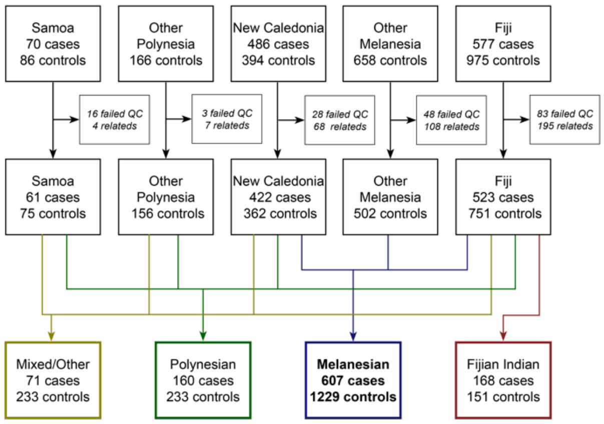

C

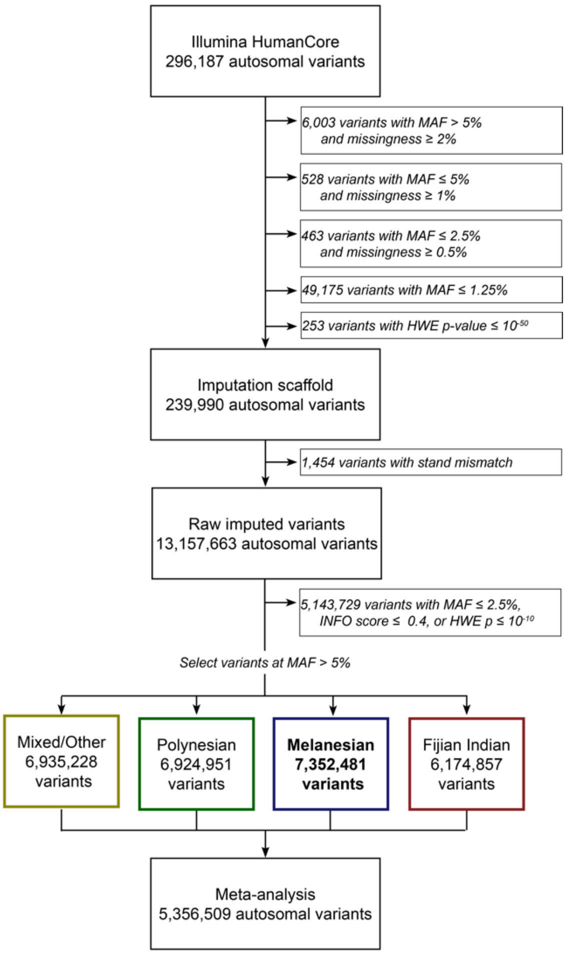

Supplementary Figure 1 | Phenotype of cases and outcome of quality control procedures. A, Phenotypic characteristics of cases recruited by the Pacific Islands Rheumatic Heart Disease Genetics Network. Severity was based on phenotypic findings. **B**, Selection of samples and allocation to ancestral strata. **C**, Selection of variants used for imputation and calculation of the kinship matrix. MAF, minor allele frequency; HWE, Hardy-Weinberg Equilibrium; INFO, imputation information metric[31].

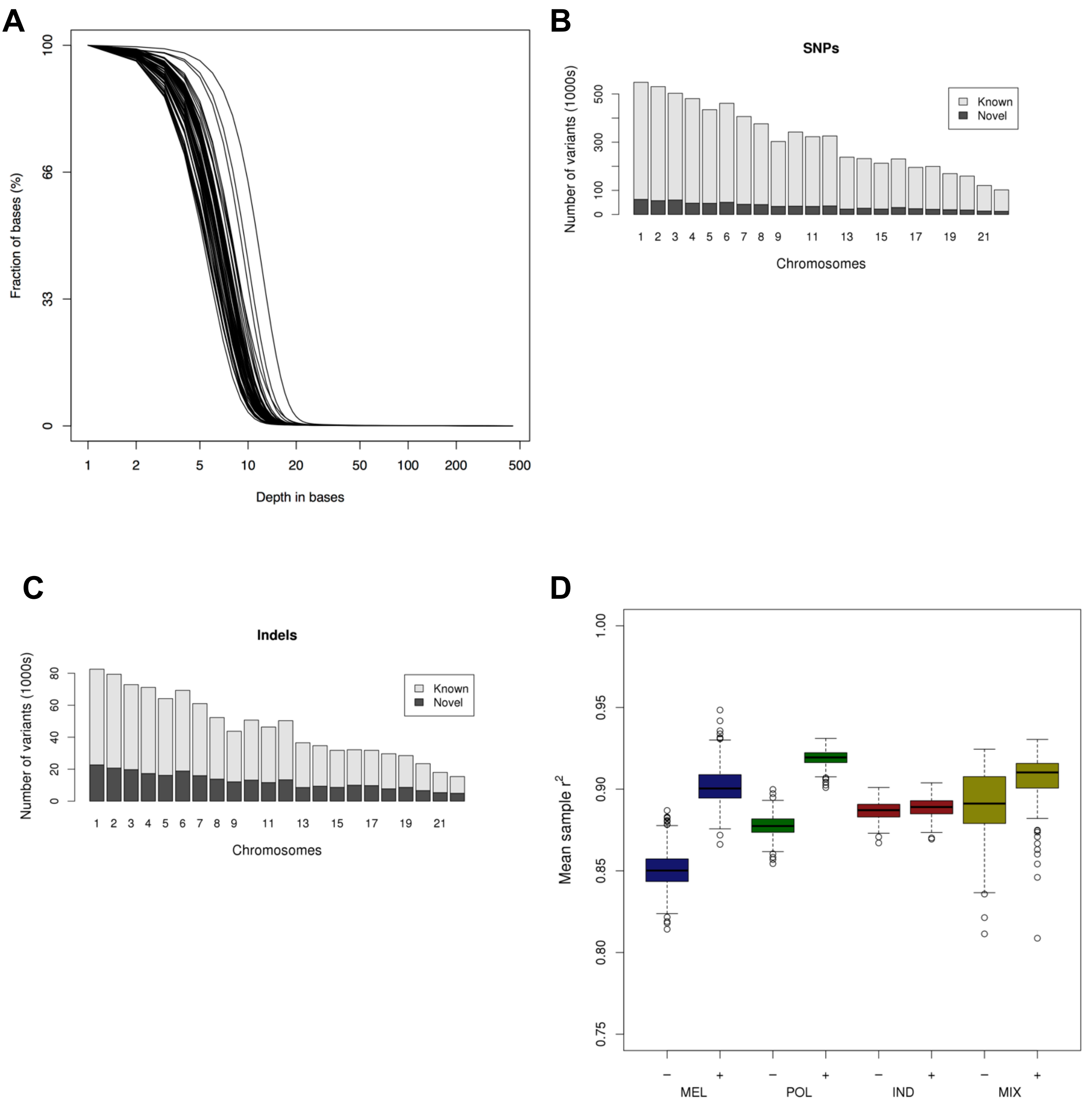

Supplementary Figure 2 | Low-coverage whole genome sequencing of the population-specific imputation panel and imputation accuracy with and without sequence data. A, The lines show, for each of sixty-four samples, the fraction of bases covered at a given depth on a logarithmic scale. The bar charts show the proportions of, **B**, single nucleotide polymorphisms (SNPs) and, **C**, insertion-deletion variants (INDELs) captured in the whole-genome sequencing by chromosome that are known and novel. Novel refers to variants not recorded in the NCBI database of short genetic variations (dbSNP). **D**, For each self-reported ancestral population, a boxplot is shown for mean sample concordance for variant of all frequencies following imputation of chromosome 1 carried out with (+) and without (-) the population-specific low coverage sequence data as a reference panel. Samples that had been sequenced were excluded from this analysis. MEL, Melanesians; POL, Polynesian; IND, Fijian Indian; MIX, Mixed and other.

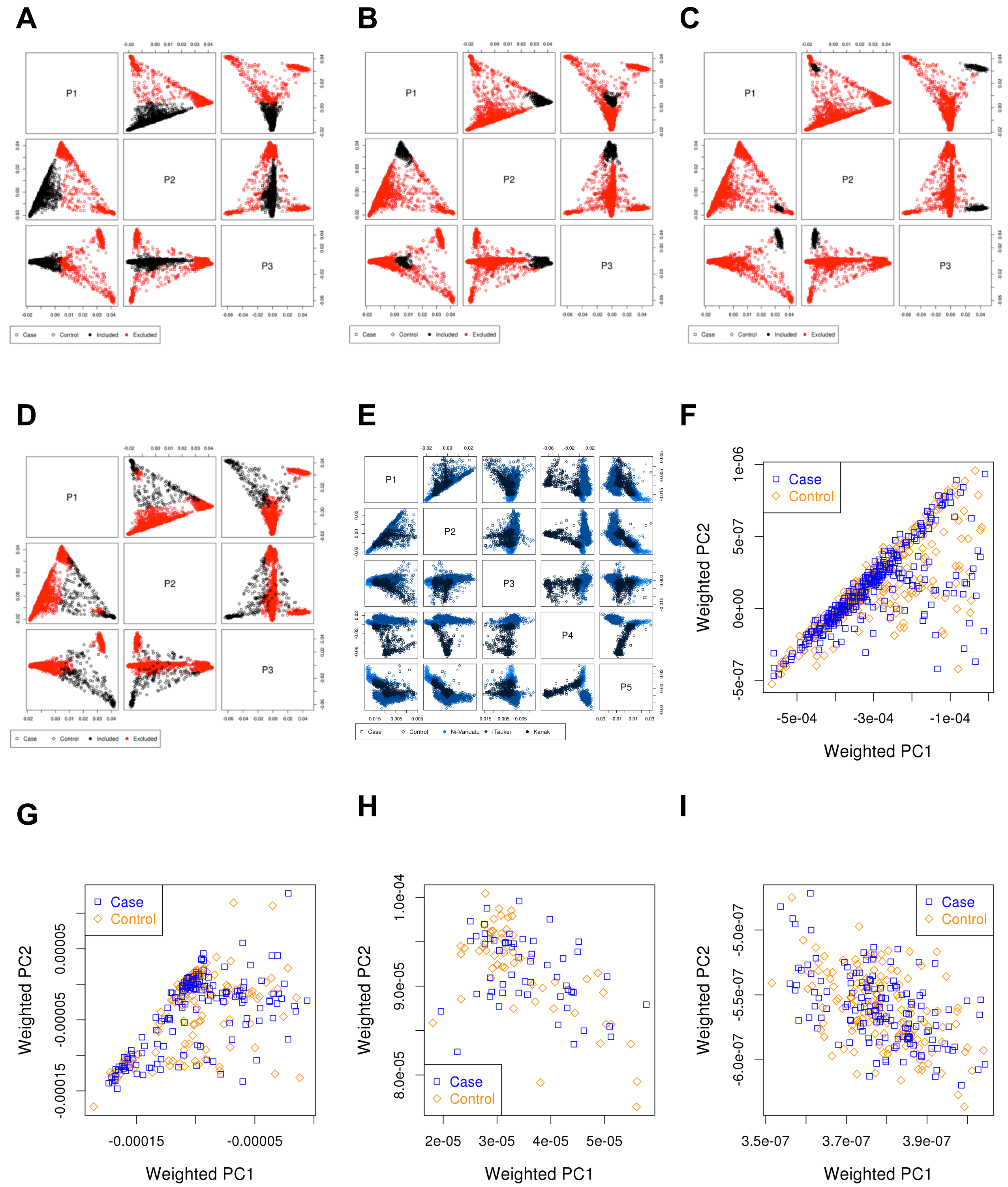
A
B
C
P1
P2
P3
Case
Control
Included
Excluded
D
E
F
P4
P5
Ni-Vanuatu
iTaukei
Kanak
Weighted PC1
Weighted PC2
1e-06
5e-07
0e+00
-5e-07
-5e-04
-3e-04
-1e-04
G
H
I
0.00005
-0.00005
-0.00015
-0.00015
-0.00005
1.0e-04
9.0e-05
8.0e-05
2e-05
3e-05
4e-05
5e-05
-5.0e-07
-5.5e-07
-6.0e-07
3.5e-07
3.7e-07
3.9e-07

Supplementary Figure 3 | Principal components analysis to define ancestral strata and subsets of matched case-control pairs. To illustrate the ancestral strata, plots of the first three principal components (PCs) are shown for **A,** Melanesian, **B,** Polynesian, **C,** Fijian Indian, and **D,** Mixed and other. In each stratum, included individuals are coloured black while excluded individuals are coloured red. To investigate residual population structure in the Melanesian stratum, plots of the first five PCs are shown in **E**, with cases indicated by empty squares and controls indicated by empty diamonds. Self-reported ancestry is indicated by three shades of blue: Ni-Vanuatu by the lighter shade, iTaukei by the medium shade and Kanak by the darkest shade (the major indigenous peoples of Vanuatu, Fiji and New Caledonia respectively). To illustrate the ancestry matched case-control pairs, plots of the first two PCs, weighted by how much of phenotypic variance each explained in multiple regression, are shown for pairs reporting, **F**, iTaukei ancestry, **G**, Kanak ancestry, **H**, Samoan ancestry, and, **I**, Fijian Indian ancestry. Cases coloured blue and controls coloured orange. In all nine plots, cases are indicated by empty squares and controls indicated by empty diamonds.

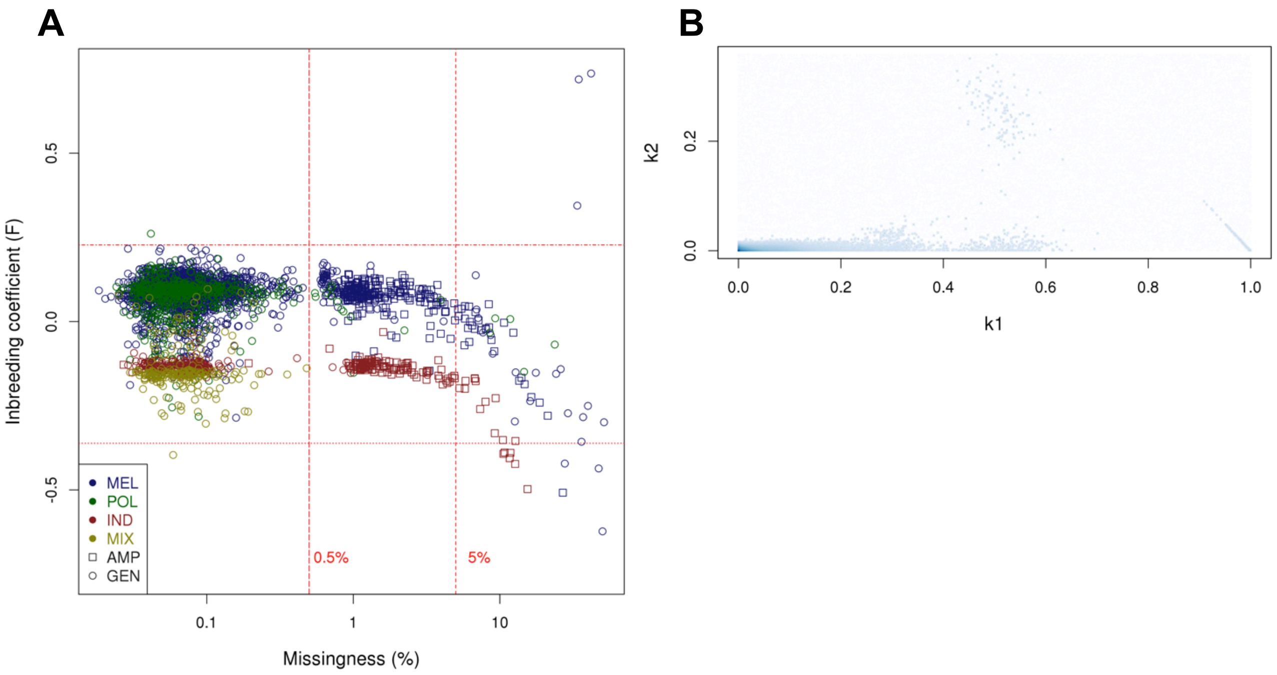

Supplementary Figure 4 | Assessment of heterogeneity and relatedness in the study. A, Autosomal homozygosity is plotted against missingness on a logarithmic scale by self-reported ancestry and the nature of the DNA sample. Horizontal lines are drawn at three standard deviations from the mean of autosomal homozygosity amongst individuals with missingness less than 5% reporting Melanesian/Polynesian ancestry or Fijian Indian ancestry. The Melanesian classification includes individuals reporting iTaukei, Kanak or Ni-Vanatuan ancestry. **B**, Pairwise relatedness estimates are plotted: k1 is an estimate of the fraction of sites where two individuals share one allele identity by descent (IBD) and k2 is the fraction of sites where two alleles are shared. The density of points is related to the colour scale blue to white using an exponent of 0.09 where dark blue represents the densest regions of the plot. Of 5839653 pairwise estimates, 310,024 (5.0%) had relatedness greater than 5% where $r = 0.5\text{k1} + \text{k2}$. MEL, Melanesians; POL, Polynesian; IND, Fijian Indian; MIX, Mixed and other; AMP, genome-wide amplified sample; GEN, genomic sample.

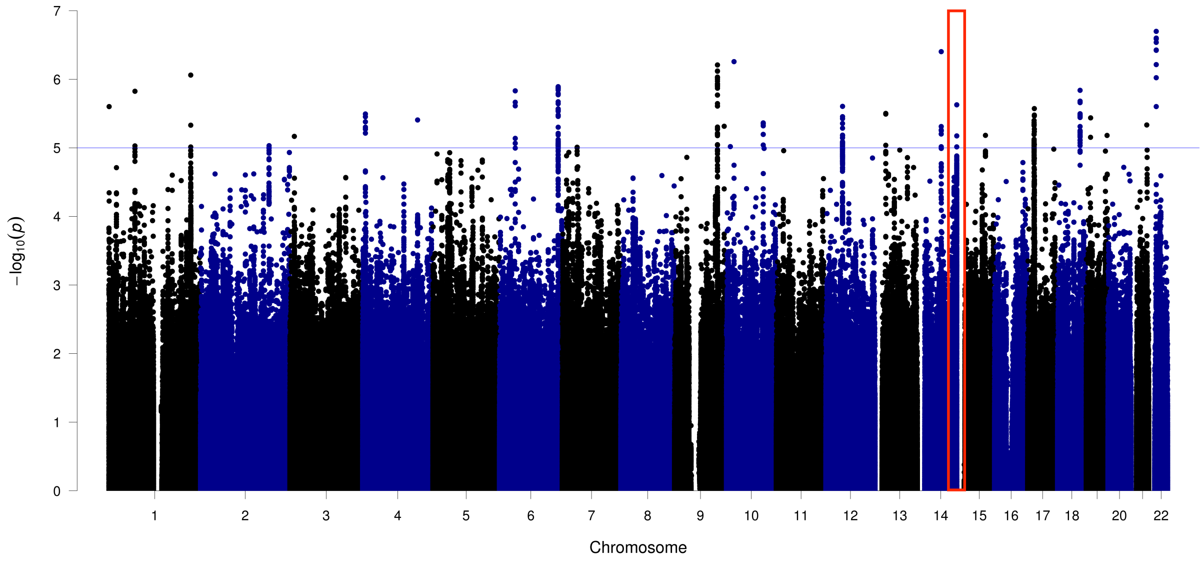

Supplementary Figure 5 | Genome-wide discovery analysis for RHD susceptibility. For each variant, the negative common logarithm of the p-value from the LMM analysis is plotted against genomic position. The blue horizontal line indicates suggestive significance (LMM, $p=10^{-5}$). The signal located in the immunoglobulin heavy chain (IGH) locus is framed with a red box.

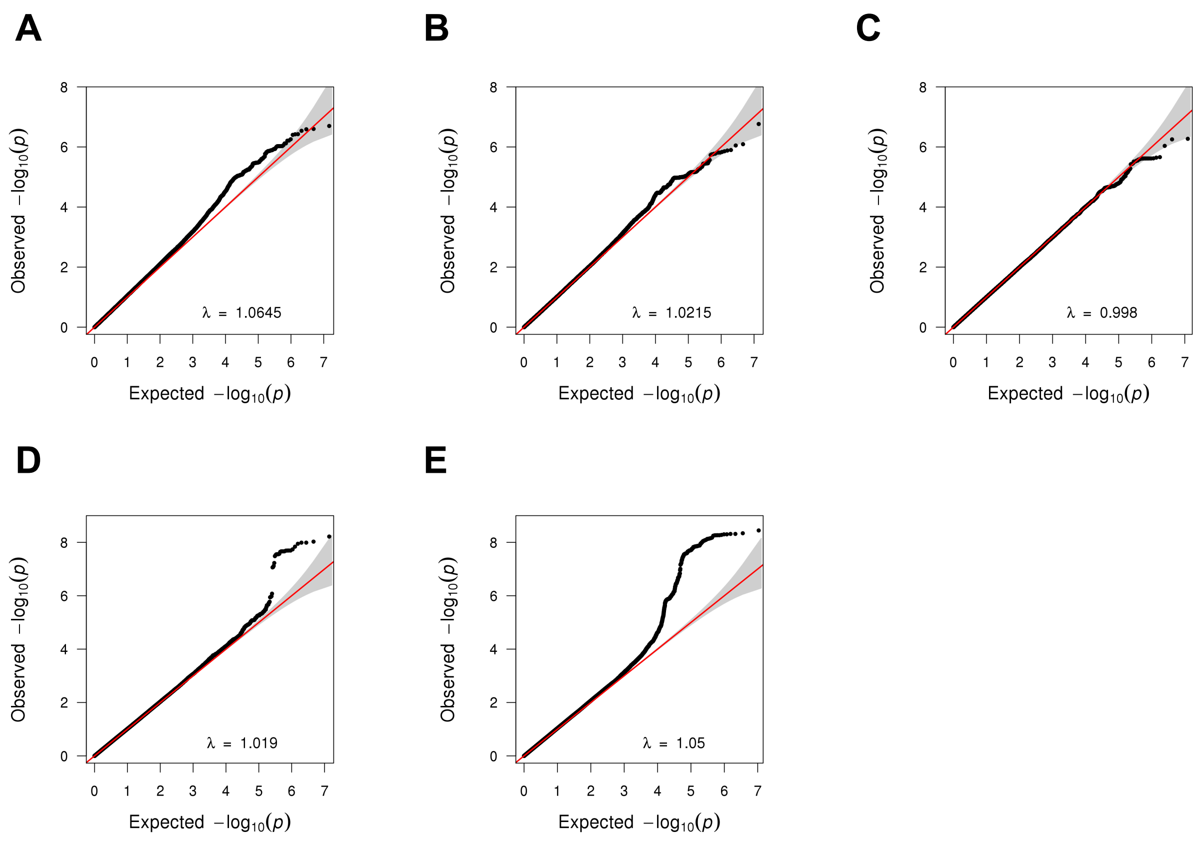

Supplementary Figure 6 | Quantile-quantile plots for primary, replication and meta-analyses. Quantile-quantile (QQ) plots are shown for the LMM analyses in, **A**, Melanesian, **B**, Polynesian, **C**, Fijian Indian, **D**, Mixed or other strata, and in, **E**, for the inverse-variance weighted fixed effects meta-analysis limited to variants present with MAF exceeding 5% in all four strata. Each point represents an individual variant. An estimate of the genomic inflation factor (λ) is shown.

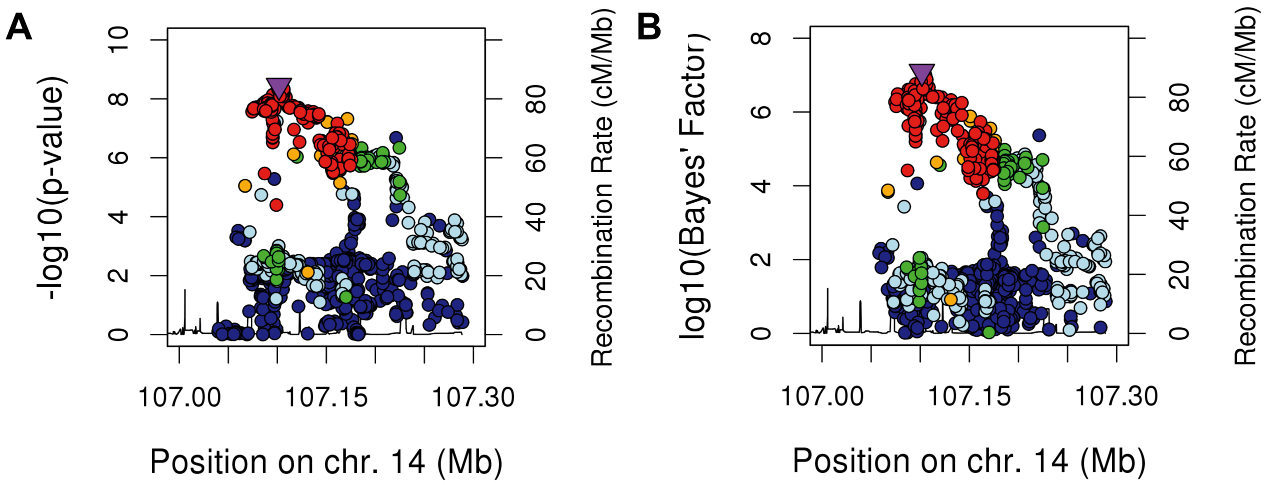

C

Gene segment	SNP	Genomic position	HGVS notation	P_{FE}*	$log_{10}BF$†	VEGA residue	IMGT residue	Amino acids	PolyPhen score‡	IMGT allele
IGHV4-61	rs202117805	107095296	c.184C>G	7.40×10^{-9}	6.76	62	46	Ala/Pro	0.756	02
	rs200931578§	107095268	c.212A>G	1.87×10^{-7}	5.53	71	55	Arg/Tyr	0.000	02
	rs202166511§	107095269	c.211T>C	1.54×10^{-7}	5.52					
	rs201076896§	107095259	c.221A>C	2.10×10^{-7}	5.20	74	58	Thr/Tyr	0.239	02
	rs201691548§	107095260	c.220T>A	2.01×10^{-7}	5.35					
IGHV1-58	rs1858692	107078790	c.19G>A	1.91×10^{-8}	6.23	7	N/A¶	Val/Ile	0.011	N/A¶

*Fixed-effects p-value from the genome-wide meta-analysis. †Common logarithm of the Bayes factor from the Bayesian trans-ancestral meta-analysis. ‡Polyphen-2 score based on the method outlined in Adzhubei et al (2010). §Both rs200931578/rs202166511 and rs201076896/rs201691548 are double nucleotide polymorphisms each resulting in a single amino acid change. ¶The rs1858692 variant localises to the leader sequence of IGHV1-58 which is not present in the final heavy domain. HGVS, Human Genome Variation Society; IMGT, International Immunogenetics Information System; VEGA, Vertebrate Genome Annotation database (vega.sanger.ac.uk/)

Supplementary Figure 7 | Frequentist and Bayesian refinement of the immunoglobulin heavy chain locus signal. For an interval stretching 150kb either side of the lead variant, genomic position is plotted against: **A**, the negative common logarithm of the p-value from the meta-analysis; **B**, the common logarithm of the Bayes' factor from the trans-ancestral meta-analysis. The most associated variant (rs11846409) is represented by a purple triangle. Other variants are coloured by linkage disequilibrium (LD) with that variant averaged across the entire dataset (estimated r^2: dark blue, 0-0.2; light blue, 0.2-0.4; green, 0.4-0.6; yellow, 0.6-0.8; red, 0.8-1.0). The recombination rate is shown as a line plotted on the right-hand y-axis. These plots are based on those drawn by the widely used LocusZoom software. In **C**, details of the five missense variants in the 99% credible set from the Bayesian analysis are shown.

A

	Forward and Reverse Primers
Polymerase Chain Reaction	**F** CAA TGC AGT AGA TTC CAA GGT TAG A<br>**R** TTC ACC TCT CCG TAC AAA GGC
Chain Termination Sequencing	**F** TGG TGA CTC GAC TCT TGA GG<br>**R** CAC CAC CCA CAT GCA AAT CC

B

	Optimised Polymerase Chain Reaction Procedures	
Cycle	Initial denaturation 95°C for 15 mins	
	Denaturation 94°C for 30 secs	44 cycles
	Annealing 65.5°C for 30 secs	44 cycles
	Extension 72°C for 80 secs	44 cycles
	Final extension 72°C for 10 mins	
Conditions	Template 2.5 ng/μl<br>$MgCl_2$ 0.6mM<br>DMSO 3%	

Supplementary Figure 8 | Details of Polymerase Chain Reaction and Chain Termination Sequencing. **A**, Primers used for the amplification and sequencing of the *IGHV4-61* locus. **B**, Optimised conditions used for polymerase chain reactions.

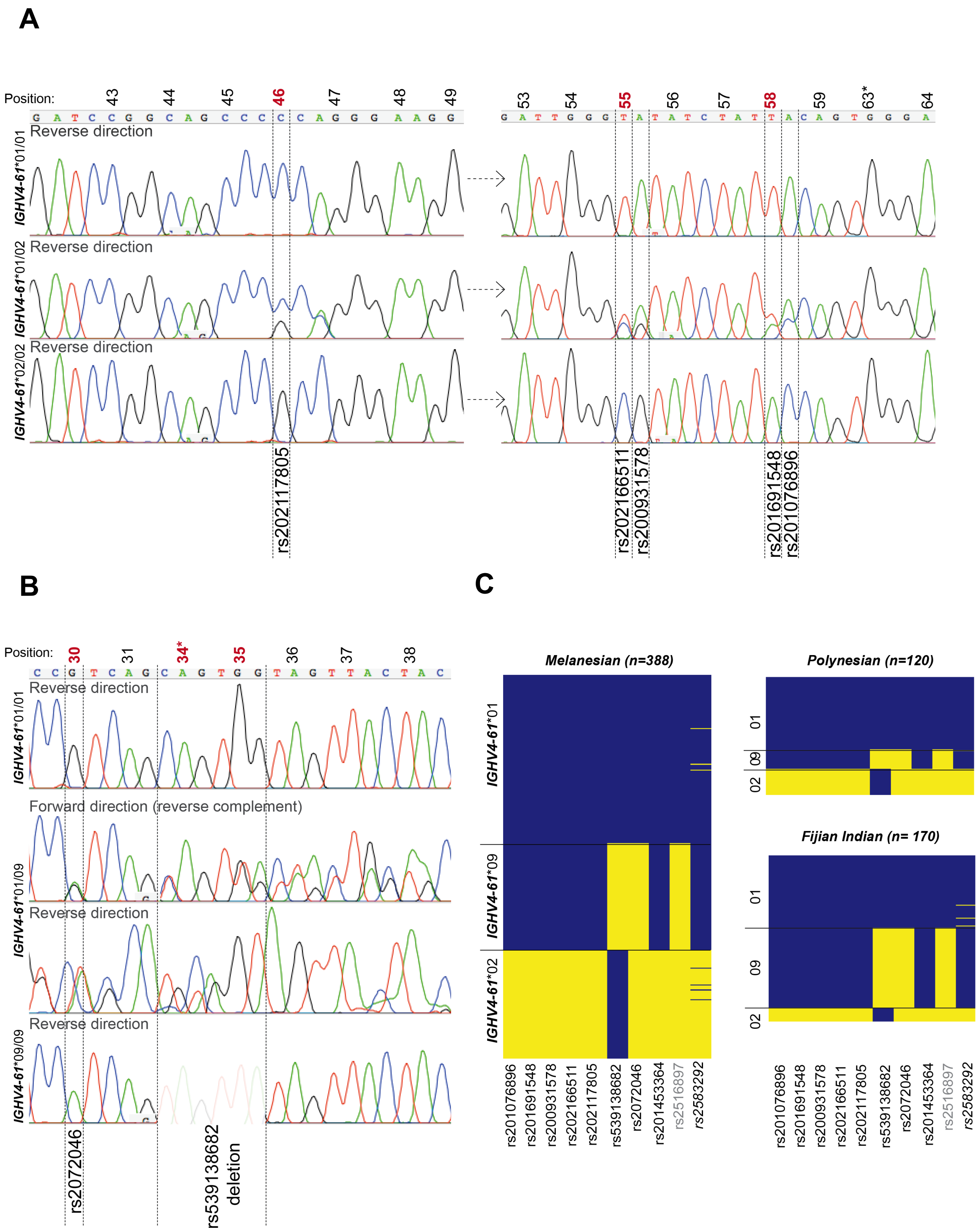
A
Position:
43
44
45
46
47
48
49
53
54
55
56
57
58
59
63*
64
Reverse direction
IGHV4-61*01/01
IGHV4-61*01/02
IGHV4-61*02/02
rs202117805
rs202166511
rs200931578
rs201691548
rs201076896
B
Position:
30
31
34*
35
36
37
38
Reverse direction
Forward direction (reverse complement)
IGHV4-61*01/01
IGHV4-61*01/09
IGHV4-61*09/09
rs2072046
rs539138682
deletion
C
Melanesian (n=388)
Polynesian (n=120)
Fijian Indian (n= 170)
IGHV4-61*01
IGHV4-61*09
IGHV4-61*02
01
09
02
rs201076896
rs201691548
rs200931578
rs202166511
rs202117805
rs539138682
rs2072046
rs201453364
rs2516897
rs2583292

Supplementary Figure 9 | Haplotypes at the *IGHV4-61* locus in the study population. Representative chromatograms from individuals with two copies of the *IGHV4-61**01 allele compared to those with **A**, one or two copies of *IGHV4-61**02 allele, with the five variants from the 99% credible set annotated, and **B**, one or two copies of the novel haplotype (provisionally designated *IGHV4-61**09) comprising a six base in-frame deletion (rs539138682*)* and a missense variant (rs2072046), together converting the sequence of *IGHV4-61* to that of *IGHV4-59*. Notably, we find the in-frame deletion has dramatically different minor allele frequency (207 of 678 chromosomes, 30.5%) compared to that reported by the 1000 Genomes Project who submitted the variant to dbSNP (3 of 5008 chromosomes, 0.06%). Above the chromatograms, codons are numbered using IMGT unique notation with variable residues in red; in places this numbering is interrupted (asterisked) because variable domains have different length complementary determining regions. In **C**, the three *IGHV4-61* haplotypes are shown by ancestry for the 339 Sanger-sequenced individuals (678 chromosomes) included in the association analyses. Haplotypes run horizontally with reference and non-reference alleles coloured blue and yellow, respectively. In addition to the seven coding variants, the haplotypes extend to a synonymous variant in the leader sequence of *IGHV4-61* (rs2516897) as well as the nearest (9kb upstream) and most strongly associated (FE meta-analysis, $p=5.5x10^{-9}$) directly genotyped variant (rs2583292), which is in strong linkage disequilibrium with *IGHV4-61**02 (r^2=0.94).

A

Part	Accession	Allele	Sequence
L-PART1	AH007113	61*01	ATGAAACACCTGTGGTTCTTCCTCCTCCTGGTGGCAGCTCCCAGAT
	L10097	61*02	........T.....................................
	KX389267	61*09	........T..............T......................
	AB019438	59*01	........T..............T......................
L-PART2	AH007113	61*01	GGGTCCTGTCC
	L10097	61*02	....
	KX389267	61*09	....
	AB019438	59*01	....
FR1-IMGT	AH007113	61*01	CAGGTGCAGCTGCAGGAGTCGGGCCCAGGACTGGTGAAGCCTTCGGAGACCCTGTCCCTCACCTGCACTGTCTCT
	L10097	61*02	....AC.............................
	KX389267	61*09	....
	AB019438	59*01	....
CDR1-IMGT	AH007113	61*01	GGTGGCTCCGTCAGCAGTGGTAGTTACTAC
	L10097	61*02	.........A....................
	KX389267	61*09	.........A....------..........
	AB019438	59*01	.........A....------..........
FR2-IMGT	AH007113	61*01	TGGAGCTGGATCCGGCAGCCCCCAGGGAAGGGACTGGAGTGGATTGGGTAT
	L10097	61*02	.....................G.C........................CG.
	KX389267	61*09	....
	AB019438	59*01	....
CDR2-IMGT	AH007113	61*01	ATCTATTACAGTGGGAGCACC
	L10097	61*02	......AC.............
	KX389267	61*09	....
	AB019438	59*01	....
FR3-IMGT	AH007113	61*01	AACTACAACCCCTCCCTCAAGAGTCGAGTCACCATATCAGTAGACACGTCCAAGAACCAGTTCTCCCTGAAGCTGAGCTCTGTGACCGCTGCGGACACGGCCGTGTATTACTGT
	L10097	61*02	....C..A.....................
	KX389267	61*09	....C..A.....................
	AB019438	59*01	....
CDR3-IMGT	AH007113	61*01	GCGAGAGA
	L10097	61*02	....
	KX389267	61*09	......C.
	AB019438	59*01	....

B

Part	Accession	Allele	Sequence
FR1-IMGT	AH007113	61*01	QVQLQESGPGLVKPSETLSLTCTVS
	L10097	61*02	...............Q.........
	KX389267	61*09	....
	AB019438	59*01	....
CDR1-IMGT	AH007113	61*01	GGSVSSGSYY
	L10097	61*02	...I......
	KX389267	61*09	...I--S...
	AB019438	59*01	...I--S...
FR2-IMGT	AH007113	61*01	WSWIRQPPGKGLEWIGY
	L10097	61*02	.......A........R
	KX389267	61*09	....
	AB019438	59*01	....
CDR2-IMGT	AH007113	61*01	IYYSGST
	L10097	61*02	..T....
	KX389267	61*09	....
	AB019438	59*01	....
FR3-IMGT	AH007113	61*01	NYNPSLKSRVTISVDTSKNQFSLKLSSVTAADTAVYYC
	L10097	61*02	....
	KX389267	61*09	....
	AB019438	59*01	....
CDR3-IMGT	AH007113	61*01	AR
	L10097	61*02	..
	KX389267	61*09	..
	AB019438	59*01	..

Supplementary Figure 10 | Nucleotide and amino acid changes in the coding region of the *IGHV4-61* locus. Base pair changes in the coding regions of *IGHV4-61**02, *IHGV4-61**09 and *IGHV4-59**01 are shown in relation to *IGHV4-61**01 as **A**, nucleotide sequence, and **B**, amino acid sequence. The sequence is divided using IMGT notation.

```
      1       10        20        30        40        50        60        70        80        90       100       110       120       130       140
61*01 TCACCTCTCCGTACAAAGGCACCACCCACATGCAAATCCTTACTTAAGCACCCACAGGAAACCACCACACATTTCCTTAAATTCAGGTTCCAGCTCACATGGGAAATACTTTCTGAGAGTCCTGGACCTCCTGTGCAAGA
61*02 ........................................C.............................................................A.....................................
61*09 ........................................C..............................................G....................................................
59*01 ..........A.............................C...................................................................................................

             150       160       170       180       190       200       210       220       230       240       250       260       270       280
61*01 ACATGAAACACCTGTGGTTCTTCCTCCTCCTGGTGGCAGCTCCCAGATGTGAGTGTCTCAGGGATCCAGACATGGGGGTATGGGAGGTGCCTCTGATCCCAGGGCTCACTGTGGGTCTCTCTGTTCACAGGGGTCCTGTC
61*02 ..........T..................................................................A.......C..........G..T........................................
61*09 ..........T..............T...................................................A..............................................................
59*01 ..........T..............T............................A......................A..............................................................

             290       300       310       320       330       340       350       360       370       380       390       400       410       420
61*01 CCAGGTGCAGCTGCAGGAGTCGGGCCCAGGACTGGTGAAGCCTTCGGAGACCCTGTCCCTCACCTGCACTGTCTCTGGTGGCTCCGTCAGCAGTGGTAGTTACTACTGGAGCTGGATCCGGCAGCCCCCAGGGAAGGGAC
61*02 .............................................AC......................................A.........................................G.C..........
61*09 .....................................................................................A...------.............................................
59*01 .....................................................................................A...------.............................................

             430       440       450       460       470       480       490       500       510       520       530       540       550       560
61*01 TGGAGTGGATTGGGTATATCTATTACAGTGGGAGCACCAACTACAACCCCTCCCTCAAGAGTCGAGTCACCATATCAGTAGACACGTCCAAGAACCAGTTCTCCCTGAAGCTGAGCTCTGTGACCGCTGCGGACACGGCC
61*02 ..............CG.......AC..............................................................................................C..A.........
61*09 ...............................................................................................................................C..A.........
59*01 ............................................................................................................................................

             570       580       590       600       610       620       630       640       650       660       670       680       690       700
61*01 GTGTATTACTGTGCGAGAGACACAGTGAGGGGAGGTGAGTGTGAGCCCAGACACAAACCTCCCTGCATGGACGCGGAGGGGACCGGCGCAGGTGCTGCTCAGGACCAGCAGGTGGCGCGCGGGGCCCCCAGAGCATGAGG
61*02 .....................................................A.............G...G.........G.G.C.............T..CG........G..............A............
61*09 ..................C..........................G.......A.............G...G.........G.G.C.........A................G.........C....A............
59*01 .....................................................A........G....G...G..............................CG........G..............A.......G....

             710       720       730       740       750       760       770       780       790       800       810       820       830       840
61*01 CCGGGTCAGGAGCAGGTGCAGGGAGGGCGGGGCTTCCTCATCTGCTCAGTGGTCTCCGTCCTCGCCAGCACCTC-GCTGTCACCAGGGCTCCTCTTTCTTTATTATCTGTGGTTCTGCTTCCTCACATTCTTGTGCCAGG
61*02 ..C....T.......................................T.........C................-.................................................................
61*09 ..........................................................................-.................................................................
59*01 ..C......................................................C................A........C....G.................................................A.

             850       860       870       880       890       900       910       920       930       940       950       960       970       980
61*01 AAAGAAACGAGGAAGACAAATTTTCGTCTATAGTTGAAGCTTCACCAATTACTAGGAACTTGCCTACAAGTTCCTGCATGACCCATTATAACTTATCGATTAAAAAATATATATTCTAATGCTTCTCACCATCTCTTGAT
61*02 ..........A......................................................................................A..........................................
61*09 ............................................................................................................................................
59*01 .......T.....................G.........T.....................T....G.............G.........G....CT......................C........G...........

             990      1000      1010      1020      1030      1040      1050      1060      1070      1080      1090      1100      1110      1120
61*01 TTGTATCATCAACTGAATTGTACCCTCTTTGAAATTCATATGATGAAACCTTAAATTCAATGGATCTATATTGGAATTTTAATGAAATAATTAAGGTTAAATGTGGTCATAATTGTAAGACCCTAATGCAATAGACGTGT
61*02 ............................................................................................................................................
61*09 ...........................................................G................................................................................
59*01 ....G................G....................CA.................T...G..............................................G........T.....T...C........

            1130      1140      1150      1160      1170      1180      1190      1200      1210      1220      1230      1240      1250      1260
61*01 TGTCTTTATAAGAAGAGGAAGAGACACCAGAGACCTCTCACTTTTCACGTGCAGGCAGAGAAGAGGCCATGTGGAGACATAGTGCACTAGAAGGTGGCCCAGTGCAAGCCAGGAAGAAGCCGCGCCAAGAACCAGCCCTG
61*02 ............................................................................................................................................
61*09 ..........G.....................................................................................................A...........................
59*01 C.............................................................................G..A.......................................T.A..........A.....
```

```
             1270      1280      1290      1300      1310      1320      1330      1340      1350      1360      1370      1380      1390      1400
61*01   CCAGCACACTAATCTTCAACATTCAGACTGCAGAATTTTAAGAAAATCAATATTTGTTGTTTAAGCCACCCACTCCTGTTGTCTTCTTATGAAGATCCAGACAGACTAATACCACATAACTCTGTTAGTGCTGTCCCCTG
61*02   ......A.........................................TG..........C....................................................................C..........
61*09   .................................................G...C...........................................................................C..........
59*01   ....A...T.G......................................................................................................................C..........

             1410      1420      1430      1440      1450      1460      1470      1480      1490      1500      1510      1520      1530      1540
61*01   GATGGAGAATTAGCCTCCTGAGGCTGGGCACATCTCTCAGATTTCCACATAAAACAGGTAAAAAATAGTAGTTCTGATATAAAAACTTGTCATGTCCCTGTTGGCCAATTTCTGGGCAAGGTCTTTTAAATAAGCCAAGT
61*02   .....................................A...................................................................G..................................
61*09   A.....................................................T..................................................G..................................
59*01   ....C.....C....CG...G................................GT...C..........................T............................................G.....CT.G

             1550      1560      1570      1580      1590
61*01   TTGCGGGGAAATGGAGACCATATGTTTGTGGGACTCTAACCGTGGAATCTACTGCATTG
61*02   ..............---------------------------------------------
61*09   .............----------------------------------------------
59*01   GG..TTT.TC.CAA.AGTTGCC.T..ATCATTTA.TAGGA.A.AACTGA.GAACA..GA
```

Supplementary Figure 11 | Alignment of sequence surrounding *IGHV4-6101, *IGHV4-61**02, *IGHV4-61**09 and *IGHV4-59**01.** Amplification primers are shown in blue, bold and underlined. The coding sequence is underlined in black with the sequence that forms the IMGT allele highlighted in bold. The single non-coding position at which *IGHV4-61**09 matches *IGHV4-59**01 (but not *IGHV4-61**01 or *IGHV4-61**02) is highlighted red, whereas non-coding positions at which *IGHV4-61**09 matches *IGHV4-61**01 or *IGHV4-61**02 (but not *IGHV4-59**01) are highlighted grey.

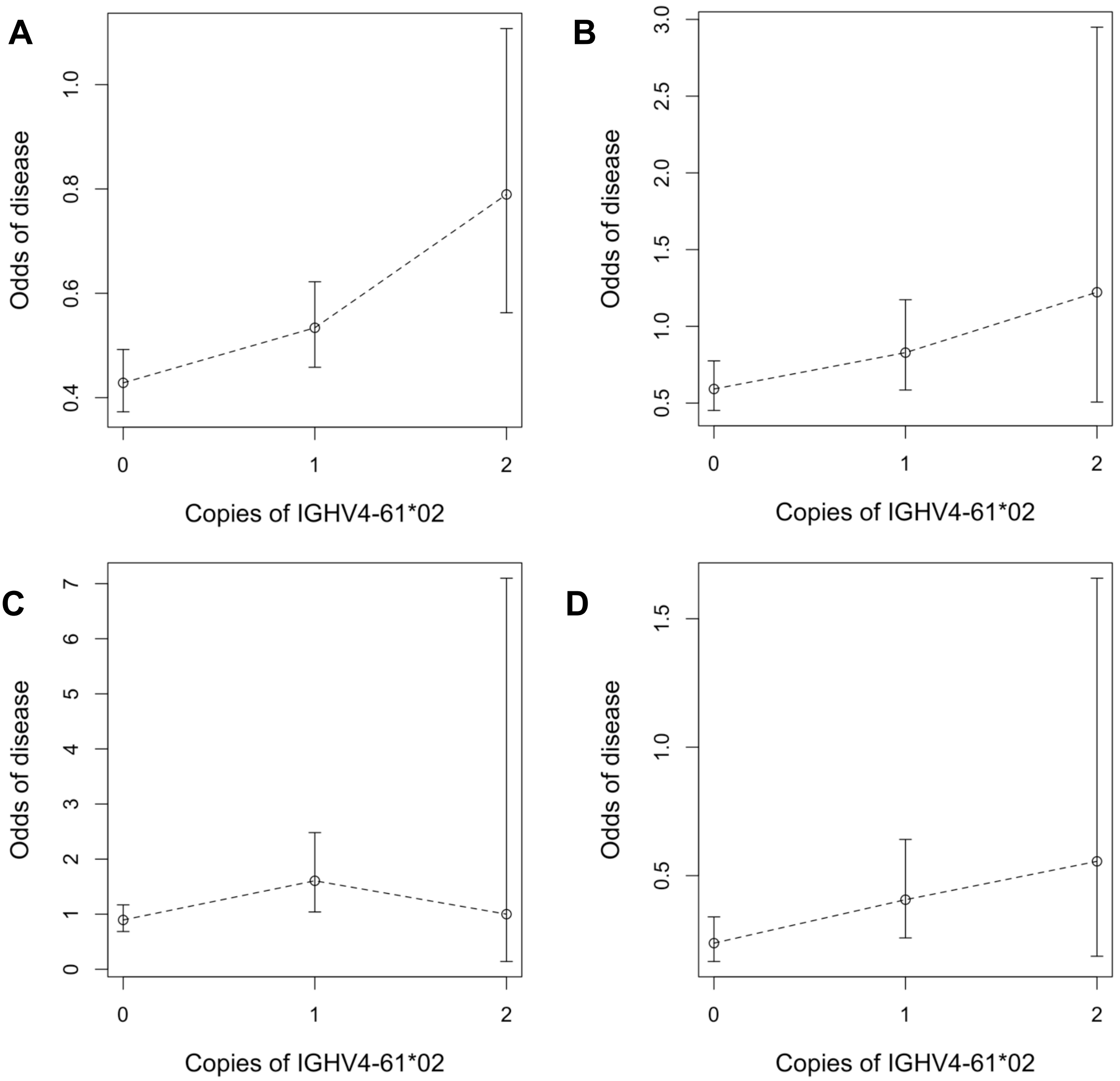

Supplementary Figure 12 | Additive effect of the *IGHV4-6102 allele on RHD susceptibility.** In **A-D**, odds of disease are plotted with 95% confidence intervals against the copies of *IGHV4-61**02 as a categorical variable based on *IGHV4-61**02 genotypes with imputation probability of 80% or more. Separate plots are shown for each ancestral strata **A**, Melanesian, **B**, Polynesian, **C**, Fijian Indian, **D**, Mixed or other.

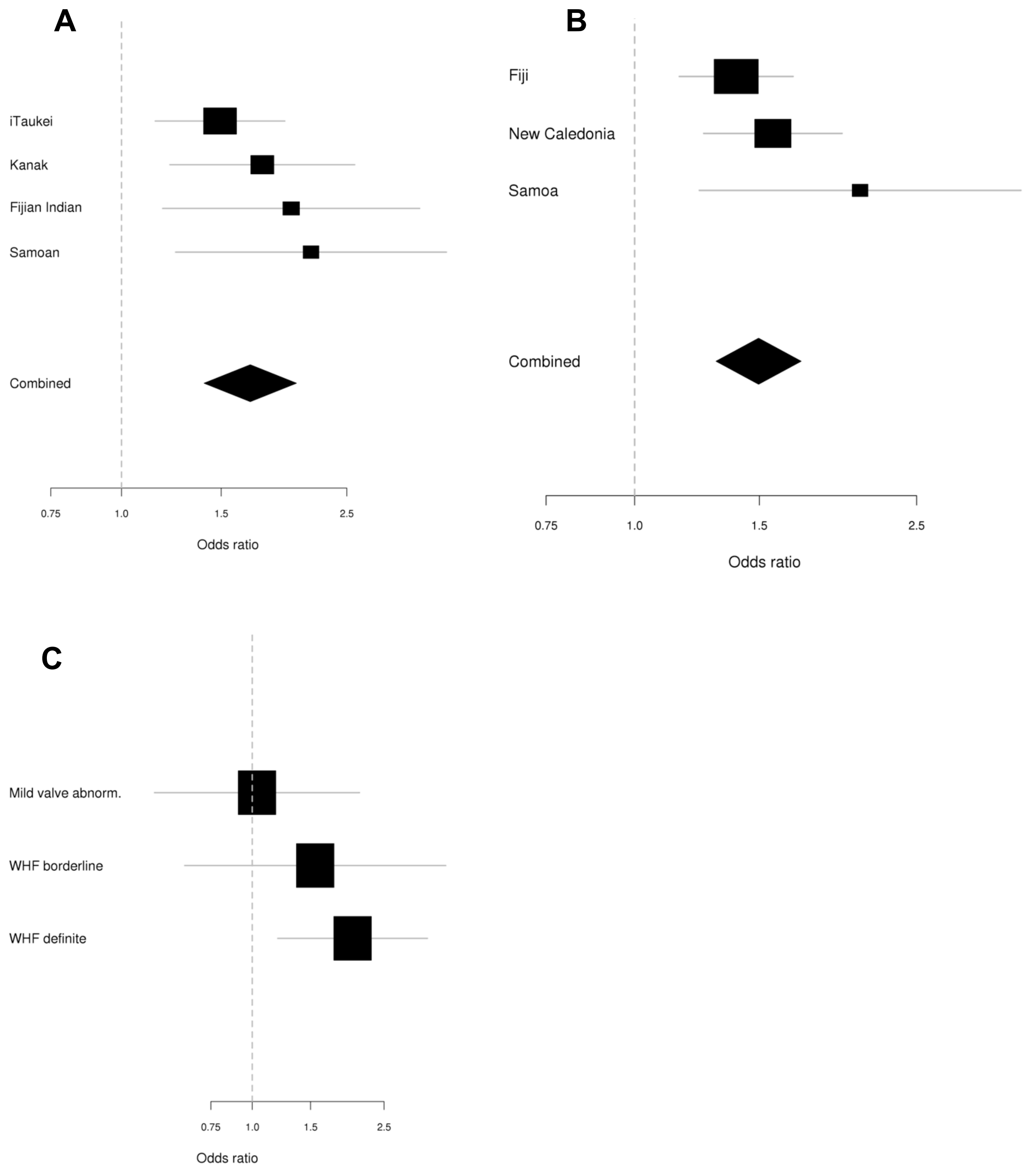
A
iTaukei
Kanak
Fijian Indian
Samoan
Combined
0.75
1.0
1.5
2.5
Odds ratio
B
Fiji
New Caledonia
Samoa
Combined
0.75
1.0
1.5
2.5
Odds ratio
C
Mild valve abnorm.
WHF borderline
WHF definite
0.75
1.0
1.5
2.5
Odds ratio

Supplementary Figure 13 | Sensitivity analyses of the effect of *IGHV4-6102 allele on RHD susceptibility.** Forest plots are shown for the effect of *IGHV4-61**02 under an additive genetic model in alternative subsets of the data: **A**, ancestry-matched case-control pairs from specific populations (see also Supplementary Figure 3); **B**, individuals of varied genetic ancestry recruited in one of the three countries from where both cases and controls were available; **C**, children recruited in Samoa with one of: non-diagnostic mild valve abnormalities, WHF borderline disease, or WHF definite disease, each compared to the Samoan controls used in the main analysis. For each analysis, the black squares center on the odds ratio estimate from LMM on a logarithmic scale and the size of the square is proportional to the analysis' weight. The horizontal line through each square corresponds to the confidence intervals (CIs). In **A-B**, the black diamond centers on the combined effect estimate by FE meta-analysis and stretches to the CIs. The dashed line indicates no effect.
